# Evaluation of Anti-Burst Performance in Mining Roadway Support System

**DOI:** 10.3390/s23020931

**Published:** 2023-01-13

**Authors:** Rupei Zhang, Siyuan Gong, Linming Dou, Wu Cai, Xuwei Li, Hui Li, Xinyuan Tian, Xiaomin Ding, Jiasheng Niu

**Affiliations:** 1 School of Mines, China University of Mining and Technology, Xuzhou 221116, China; 2 Engineering Laboratory of Mine Earthquake Monitoring, China University of Mining and Technology, Xuzhou 221116, China; 3 Dongxia Coal Mine, Huating Coal Industry Group Co., Ltd., Huating 744100, China

**Keywords:** anti-burst performance, peak particle velocity, attenuation model, numerical simulation, disaster-causing model

## Abstract

The hazardous effect of a mine earthquake on a roadway is not only related to its energy scale but also to its distance from the roadway. In this study, a signal attenuation model and a disaster-causing model were established to evaluate the mine earthquake effects based on peak particle velocity (PPV) data recorded for 37221-1 upper roadway of the Dongxia Coal Mine, China. The characteristic of dynamic loads due to mine earthquake propagation to roadway surfaces was researched, and critical PPV values were identified using FLAC^3D^ numerical simulation, which can be used to evaluate the roadway anti-burst performance under the existing support system. The results show that the support system is able to resist a mine earthquake with energy below 2.33 × 10^3^ J; however, considering the energy accumulation volume of surrounding rocks and the range of source fracture, the maximum resistible mine earthquake energy can be up to 7.09 × 10^6^ J when the roadway is 50 m away from the source. The validity and applicability of the disaster-causing models was verified by two rockburst cases that occurred during the excavation of the working face.

## 1. Introduction

Rockburst, a dynamic phenomenon where severe damage is caused by the sudden release of elastic energy accumulated in coal and rock mass, is a serious threat to a mining system due to its instantaneous explosiveness, high destructiveness, and complex diversity [1,2,3,4]. Therefore, it is necessary to apply enhanced monitoring to rockbursts and adopt certain measures to relieve or prevent their occurrences from the perspective of both safety and economy. Although rockburst research has progressed over the past decade, ineffective rock support always contributes to the toll of injuries and fatalities as a result of dynamic loading that triggers violent rock failure [5,6,7]. In recent years, due to the extensive application of fully mechanized hydraulic support, the support strength of coal mining working faces has been significantly increased, but rockburst appearances are mostly directed towards roadways from working faces, resulting in the continuous increase in frequency of rockbursts in roadways [8,9]. According to statistics, over 90% of rockbursts in coal mines occurred in roadways, which is why reinforcing roadways are of great significance for the prevention of rockburst hazards. However, not all types of roadways need high-strength support components since it is impractical to incur huge expenses reinforcing supports for roadways of shallow burial depth or low burst probability. Hence, an evaluation method regarding roadway support systems is needed to test their anti-burst performance, and then support enhancement is required for those roadways that fail to comply with the anti-burst standards to meet anti-burst requirements. With respect to the research into the evaluation of anti-burst performance of roadway support, Wang et al. [10] introduced the reliability parameter of anti-burst support design, P(x), from the perspective of flexible energy absorption and energy balance, calculated the anti-burst performance of roadway support, and discussed the feasibility of the micro-seismic reverse support parameter method. Teng et al. [11] introduced the definition of fragility and the fragility evaluation method into the roadway support system and established an evaluation index system for the fragility of roadway support systems. Liu [12] established the calculating method of anti-burst performance of roadway support systems by combining theoretical analysis with numerical simulation based on the theory of rockburst initiation. Chen et al. [13] derived the energy criteria of burst failure caused by the instability of a roadway under rockburst due to source disturbance, and proposed an evaluation method of roadway support energy under rockburst using the energy absorbed by the support system and the energy released by surrounding rocks of the roadway after the burst as the evaluation indexes. Based on the amount of rock mass yielding around the excavation (yield zone YZ-PPV criterion) and the amount of strain energy stored in the rock mass (SE-PPV criterion), Villalobos estimated the surrounding rock supporting energy capacity of six different tunnel geometries in the Diablo Regimiento Mine [14].

However, very limited studies have explored the impact of supporting structures on rockburst [15]. At the same time, few theoretical studies or evaluation methods have been found on the anti-burst performance of a roadway support system under rockburst [13]. Besides, the traditional verification methods of the support system strength and deformation resistance performance of a roadway are not all applicable to the verification of the roadway support system under rockburst that features dynamic burst load, and cannot accurately evaluate the rockburst resistance performance of the roadway support system.

Peak particle velocity (PPV) directly depends on strain and stress, and is positively related to the energy and magnitude of an earthquake, and is thus widely applied as the index of the effect of vibration on underground infrastructure [16,17,18]. In the field of coal mine safety, Mutke et al. [18] conducted an analysis on massive data about rockburst appearance and thereby found that roadways would have the probability of burst when PPV exceeded 0.05 m/s. Deng et al. [19] employed peak particle velocity (PPV) at the tunnel surface to analyze the damage to the tunnel, and found that the bolt support could greatly increase the stability of the tunnel by changing the vibration form of the particle velocity.

Therefore, this paper established a roadway disaster-causing model by adopting roadway surface PPV as the significant index to measure the anti-burst performance of a roadway on the basis of the seismic wave propagation law, and conducted a scientific evaluation on the anti-burst performance of roadways. The research provides a new method and new idea to evaluate the anti-burst performance of roadway support systems, and the research results have important practical significance for the prevention and control of rockburst.

## 2. Principle of Anti-Burst Performance Evaluation for Roadways

The occurrence of rockburst is often combined with a mine earthquake. When the seismic wave generated by the mine earthquake propagates to the near-field surrounding rock of the mining face, the tunneling face, or the roadway and the chamber in use, it will produce disturbances to the near-field surrounding rock of the mining space. Once the mechanical conditions of coal and rock mass burst are met, the rockburst will occur [20]. The generation of a mine earthquake is closely related to the fracture of the coal and rock mass. When coal and rock mass are fractured, the phenomenon of elastic energy release will occur, with seismic waves radiated outwards, and thus the accumulated damage to and the elastic energy concentration in the coal and rock mass can be described based on the energy characteristics of the seismic waves [21,22,23].

According to relevant research, a process of energy transfer exists in the period from rockburst initiation to burst appearance, and may cause the consumption and attenuation of energy [24]. Hence, the degree of damage caused to a roadway by a mine earthquake not only depends on the scale of the energy, but is also influenced by the distance from the source to the roadway, which means that the roadway will hardly be damaged even if a mine earthquake with high energy is far from it. Studying the law of seismic wave propagation and attenuation in media, its dominant mechanism is the critical intermediate step in the analysis of the process of a seismic wave disaster [25]. This paper analyzed the collected mine earthquake signals and solved the source parameters using a micro-seismic monitoring system, and then established mine earthquake attenuation models based on the propagation law of seismic waves. The attenuation models created via the fitting of every mine earthquake signal can demonstrate the characteristics of dynamic load acting on the roadway surface when the mine earthquake is propagated to the roadway. In this paper, the mine earthquake peak particle velocities and energies of many attenuation models were used in regression analysis and integrated as a roadway disaster-causing model, where the mine earthquake energy is an independent variable and the distance from the source to the roadway is a dependent variable, with a critical PPV value that causes roadway failure.

**Figure 1:**
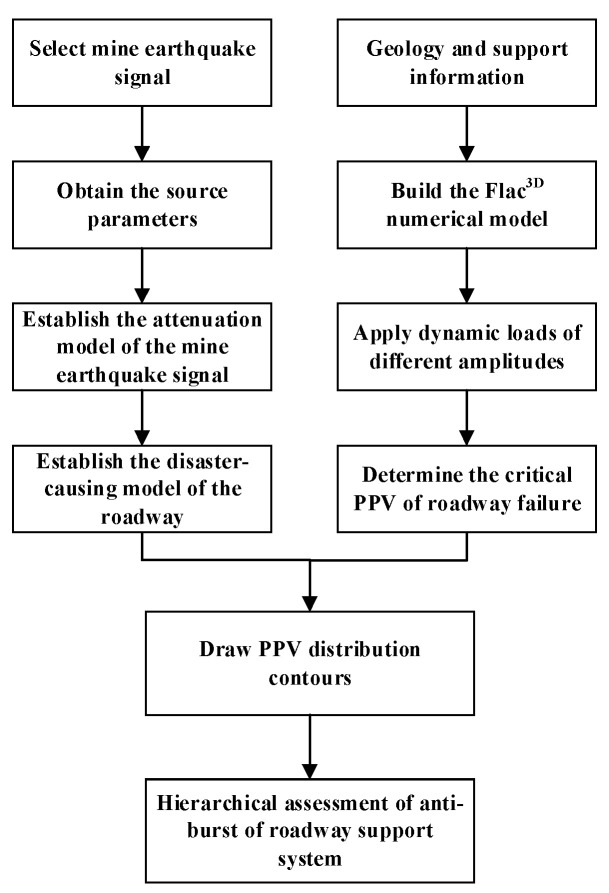
Process diagram of anti-burst performance evaluation for roadways.

The environments and wave patterns in underground mines are very complex due to many factors, such as the local site conditions, the characteristics of the source, and the medium through which waves propagate [26,27,28,29]. Therefore, it is difficult to identify the PPV value representing the propagation of dynamic load from the source to the roadway surface [30]. Fortunately, with the rapid advancement of computer technology and numerical techniques, numerical modeling is becoming an irreplaceable and indispensable method for investigating seismic wave propagation [31,32,33,34,35,36,37]. Therefore, this paper adopts the numerical simulation method to determine the critical PPV value of roadway failure by applying dynamic loads of different amplitudes. The evaluation process is shown in Figure 1, and the detailed steps are described below.

Step 1: A micro-seismic monitoring system is used to collect the seismic wave shape signals induced by mining and production, and the seismic source parameters are obtained;

Step 2: The peak particle velocity recorded by each sensor is analyzed, and the peak velocity at the source and its propagation absorption coefficient α in rock mass are calculated by the least square method according to the propagation law of vibration wave, and then the seismic attenuation model is established;

Step 3: The correlation coefficient between mine seismic energy and peak velocity was fitted to establish the tunnel disaster-causing model;

Step 4: By applying dynamic loads with different amplitudes, a numerical simulation method is used to study the damage of the roadway when the ore shock propagates to the roadway surface, so as to comprehensively determine the critical PPV at which the roadway can resist the rock.

Step 5: According to the established tunnel disaster-causing model and the critical PPV determined by numerical simulation, the PPV distribution contour nephogram is drawn to determine the maximum energy that the tunnel can resist at different distances from the focal point to the tunnel.

### 2.1. Mine Earthquake Disaster-Causing Model

The dynamic damage effect of mine earthquakes is mainly based on the residual vibration energy propagated to the surface of the stope or roadway surrounding rock. Therefore, in order to study the damage effect of seismic disturbance on the free surface of surrounding rock, it is necessary to analyze the propagation mode and attenuation characteristics of seismic waves induced by the source. In seismology, seismic energy (*E*) is deemed to be transferred by a packet of seismic waves limited in time and space to a distance (*r*) from the hypocenter [38]. In this context, after locating the mine earthquake event, its radiated energy can be calculated by building upon the time-integrated values of P-wave (*u_P_*(*t*)) and S-wave (*u_S_*(*t*)) seismograms [39]:

(1) $$E=4\pi r^{2}\rho v_{P}\int_{t_{w1}}^{t_{w2}}{\left|u_{P}\left(t\right)\right|}^{2}dt+4\pi r^{2}\rho v_{S}\int_{t_{w1}}^{t_{w2}}{\left|u_{S}\left(t\right)\right|}^{2}dt$$

where *ρ* is the density of the medium. *v_P_* and *v_S_* are the velocities of P-wave and S-wave, respectively. *t_w_*_1_ and *t_w_*_2_ are times bounding a P-wave and S-wave group. It should be noted that the energy of such motion is mostly carried by the peak particle velocity [40]. With the consideration of the damping effect of rock mass media, it can be consumed during failure through overcoming the cohesive strength of cracks or attenuated during its propagation process [41]. In field observations, the peak velocity of mine earthquake dynamic load shows a trend of power-exponent attenuation when it propagates from the source to a receiving point. The propagation law is expressed by the equation below [42,43,44]:

(2) $$\left\{ \begin{array}{l} A=A_{0}r^{-\alpha }\\ E^{\prime }=Er^{-\eta } \end{array}\right.$$

where *A* is the PPV when the mine earthquake propagation distance is *r* (m/s); *E*′ is the energy when the mine earthquake propagation distance is *r* (J); *A*_0_ is the PPV at the source (m/s); *E* is the energy at the source (J); *α* is the attenuation coefficient of peak velocity; and *η* is the energy attenuation coefficient.

In the process of the seismic wave propagation, it will cause the round-trip vibration along the equilibrium position of the particle at the focal *r_i_* and the elastic deformation of the medium, and the energy *E* at the particle is equal to the maximum kinetic energy per unit volume [45]. Therefore, the energy of the seismic wave is proportional to the square of the peak particle velocity:

(3) $$A_{0}=C\sqrt{E}$$

where *C* is the fitting coefficient between the PPV and the energy. According to relevant theoretical research and with the extensive installation of a mine micro-seismic monitoring system, the propagation law of PPV can be further expressed as [43,45]:

(4) $$A_{ij}=A_{i0}\frac{1}{r_{ij}}e^{-\alpha_{i}r_{ij}}$$

where *A_ij_* is the PPV of mine earthquake *i* recorded by sensor *j* (m/s); *A_i_*_0_ is the PPV of mine earthquake *i* at the source (m/s); *r_ij_* is the distance of mine earthquake *i* from the source to sensor *j* (m); and *α_i_* is the attenuation coefficient of peak velocity of mine earthquake *i*. By fitting the particle vibration velocity *A_ij_* of each sensor at different distances from the source, the attenuation coefficient of peak velocity *α_i_* and the peak particle velocity *A_i_*_0_ at the source can be solved, and then the attenuation model of the mine earthquake can be obtained. The roadway disaster model shown in the following equation can be composed of an attenuation model of multiple mine earthquakes:

(5) $$E={\left(\frac{A_{p}}{C}\right)}^{2}r^{2}e^{2\alpha_{0}r}$$

where *A_p_* is the critical PPV of the roadway surface resistant rockburst (m/s) and *α*_0_ is the average of all mine earthquake attenuation coefficients, i.e., $\alpha_{0}=\frac{1}{n}\sum_{i=1}^{n}\alpha_{i}$.

### 2.2. Numerical Simulation Method

#### 2.2.1. Near-Field Dynamic Load Application Method

According to the elastic wave theory, any seismic wave can be generated from a sine wave through Fourier transformation. Therefore, sine waves are the basic form of seismic waves [46]. Since the dynamic load applied to the model surface cannot immediately be propagated to the roadway surroundings, the applied dynamic load can be designed as the form of the half-sine P wave below in order to acquire the influence of the dynamic load on the roadway during the complete process:

(6) $$wave=\left\{ \begin{array}{cc} \sin\left(2\pi f*t\right) & ,t\in \left[0,\frac{1}{2f}\right]\\ 0 & ,t\in \left[\frac{1}{2f},\frac{1}{f}\right] \end{array}\right.$$

where *f* is the dynamic load frequency and thus the acting time of the dynamic load is set as 1/*f*.

According to the findings of existing research, seismic waves in a model of homogeneous rock media propagate all around from the center [47]. When the source is far from the roadway, dynamic load can be simplified as surface waves to be directly applied to the surface of the model. However, the influence of spherical waves on the model cannot be ignored when it is near the roadway. In this case, stress waves propagate the fastest to the surface area of the model closest to the source (central area), while the time it takes the stress waves to propagate to the remaining areas of the model surface is jointly determined by wave velocity and the distance between those areas and the central area. Based on this principle, the grids on the model surface are grouped by the distance to the source, and then dynamic load is applied by time-step on a basis of the arrival time of stress waves at every group.

**Figure 2:**
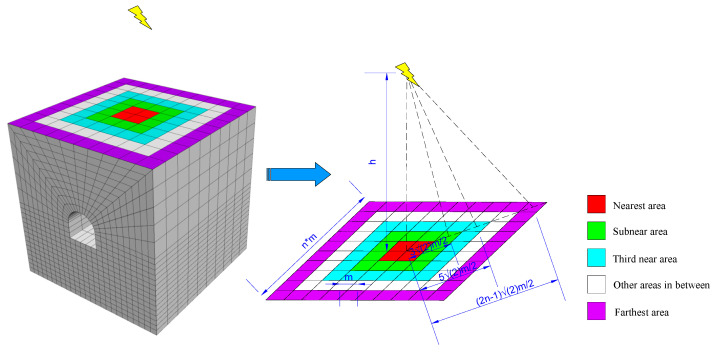
Schematic diagram of dynamic load application to model top grid.

Suppose that most of the mine earthquake is concentrated at location *h* above the center of the model, as shown in Figure 2, and the propagation velocity of seismic waves is *v*, then the dynamic load first propagates and arrives at the red area in the figure and the time of propagation is expressed as:

(7) $$t_{1}=\frac{h}{v}$$

The time of propagation to the green area is expressed as:

(8) t2=(h2+9m22)v

Likewise, the time of propagation to the farthest edge area of the top surface of the model is expressed as:

(9) tn=(h2+[(2n−1)m]22)v,n>1

**Figure 3:**
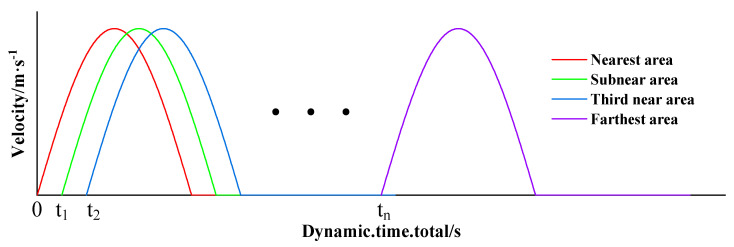
Schematic diagram of arrival time of applied dynamic load.

Figure 3 is the schematic diagram of arrival time of dynamic load at the top surface grid of the model. This paper applied dynamic load as described above in order to guarantee that the simulation results would be closer to reality.

In addition, to prevent the reflection of stress waves inside the model during the application of dynamic load, free field boundary conditions equal to the infinite model effect were introduced. Additionally, local damping of 15% was adopted.

#### 2.2.2. Simulation and Characterization Methods of Roadway Failure State

After roadway excavation begins, surrounding rocks are continuously acted on by the original in situ stress and other factors. When the deformation has accumulated enough to cause roadway failure, both the sides and roof of the roadway begin to fail. Additionally, through the analysis of multiple rockburst signals from the field, it was found that the amplitude of the seismic signals recorded by the sensors surrounding the area of rockburst was obviously higher, indicating that the burst occurrence would cause instant significant deformation and migration of the roadway area.

**Figure 4:**
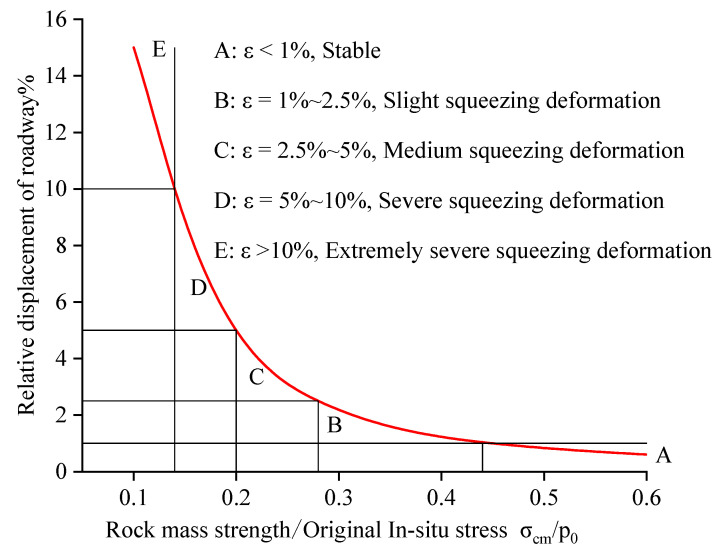
Schematic diagram of surrounding rock deformation class determination [48,49,50,51].

Aydan, Singh, and Hoek, et al. [48,49,50] proposed the deformation classification standards of surrounding rocks around roadways by combining various engineering examples and theoretical analyses based on the relative displacement of the surrounding rocks, as shown in Table 1. Among them, Hoek further classified deep roadways with large deformation using the ratio of roadway surface displacement to roadway span and the ratio of rock mass strength to maximum original in situ stress as the evaluation indexes for deep underground projects. With the combination of Table 1 and Figure 4, the relative displacement of 3% can be used as the critical value of relative deformation of roadways, i.e., it can be deemed that large roadway deformation occurs when the relative deformation of the roadway is more than 3% (the deformation of the roadway surrounding rock is greater than 3% of the roadway size) [51]. This paper adopted this value as the standard to characterize the occurrence of roadway failure.

## 3. Engineering Practice

**Figure 5:**
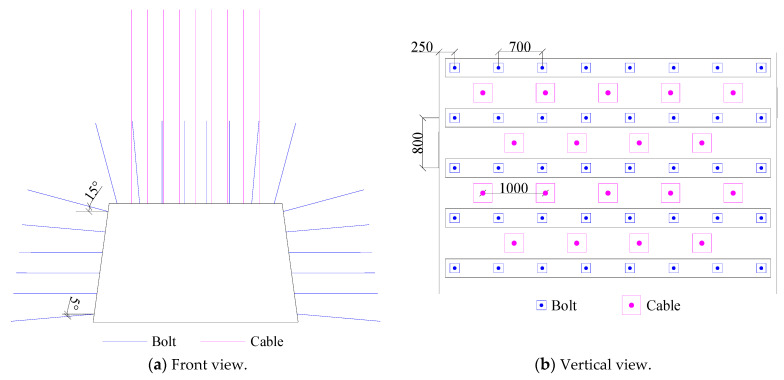
Schematic diagram of roadway support.

The 37221-1 working face of the Dongxia Coal Mine is located in the 875–940 m section of the Huating Coal Mine, with the 6-2 coal seam as the main coal seam. The elevation, maximum burial depth, and dip angle of the coal seam are 877–941 m, 570 m, and 25°–40°, respectively. The entire 37221-1 upper roadway adopted a trapezoid cross section, and was 5400 mm wide at the top, 6400 mm wide at the bottom, and 3700 mm high. Eight sets of bolts are deployed on the top of the roadway, and the row-to-row space is 700 mm × 800 mm. The top cable has a staggered arrangement, front row arrangement of five sets, rear row arrangement of four sets, and a distance of 1000 mm between one another. The parameters of the specific support form are shown in Figure 5.

The SOS micro-seismic monitoring system has been installed in the Dongxia Coal Mine for the monitoring and early warning of impact danger. The system can collect the vibration velocity signal of the rock mass through the mine earthquake picking sensor installed in the roadway floor, and then solve the source parameters, so as to realize the location and energy calculation of the mine earthquake.

### 3.1. Data Collection and Processing

**Figure 6:**
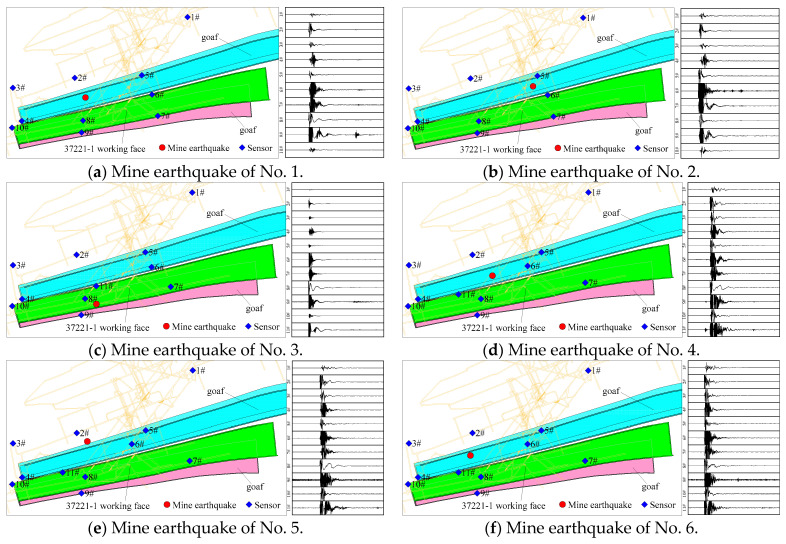
Plan of sensor layout and mine earthquake waveform.

The signals of six mine earthquakes with high energy during the production on the 37221-1 working face were selected for the establishment of the mine earthquake attenuation model for the Dongxia Coal Mine. The results of mine earthquake location, sensor layout, and waveform of each channel are shown in Figure 6.

**Figure 7:**
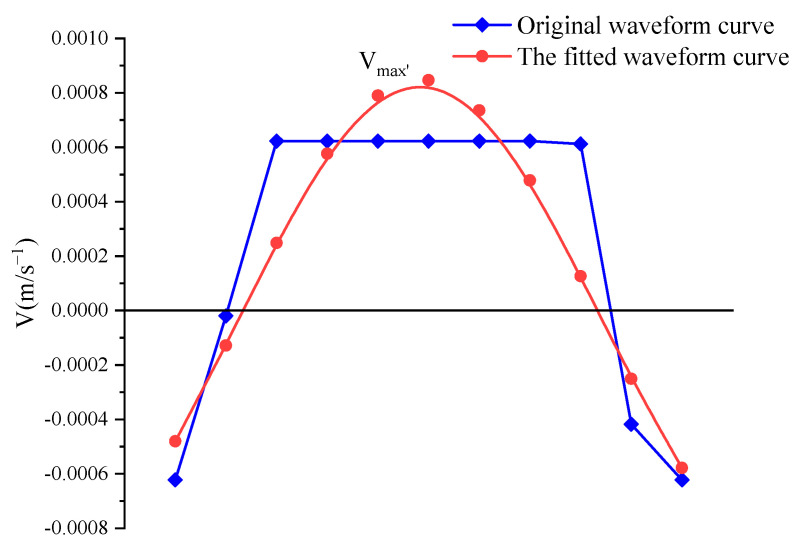
Waveform fitting diagram.

Since the waveform exceeds the range of the sensor channel, it is necessary to use a fitting method to complete the missing waveform, so as to analyze the peak particle velocity of each channel waveform. In this paper, the missing waveform is approximated piecewise by sine function, and the peak particle velocity of the waveform after fitting is the maximum value of all piecewise peak velocities. The schematic diagram of fitting principle is shown in Figure 7.

### 3.2. Disaster-Causing Model of Dongxia Coal Mine Roadway

**Figure 8:**
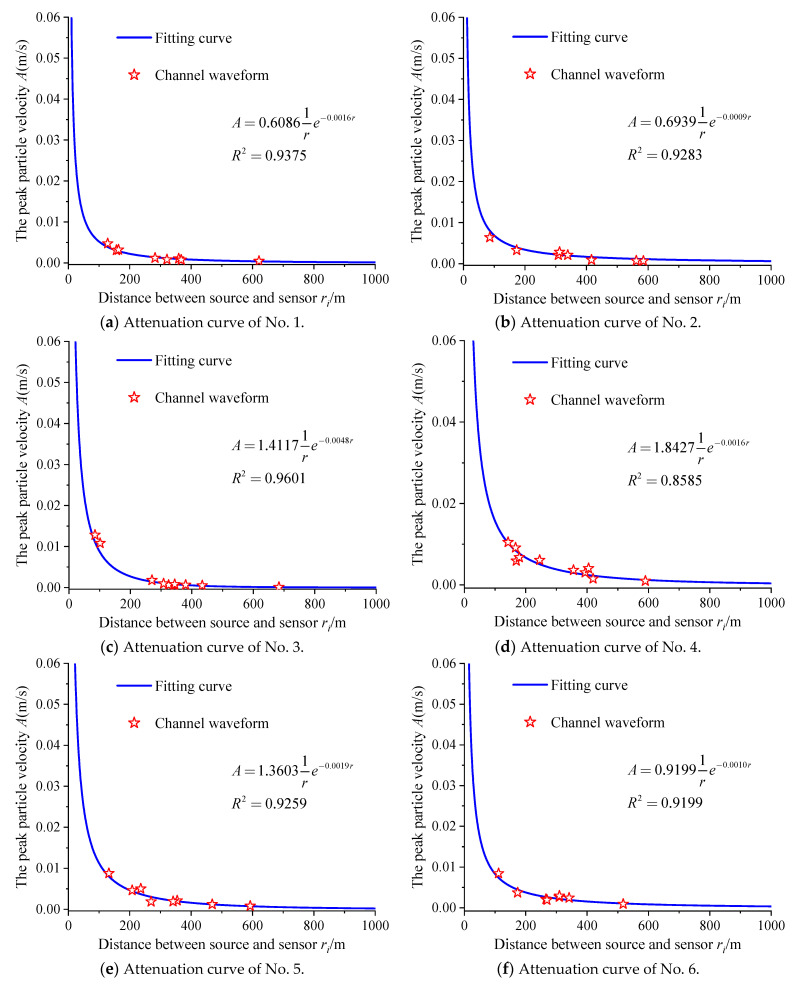
Mine earthquake attenuation curves.

**Figure 9:**
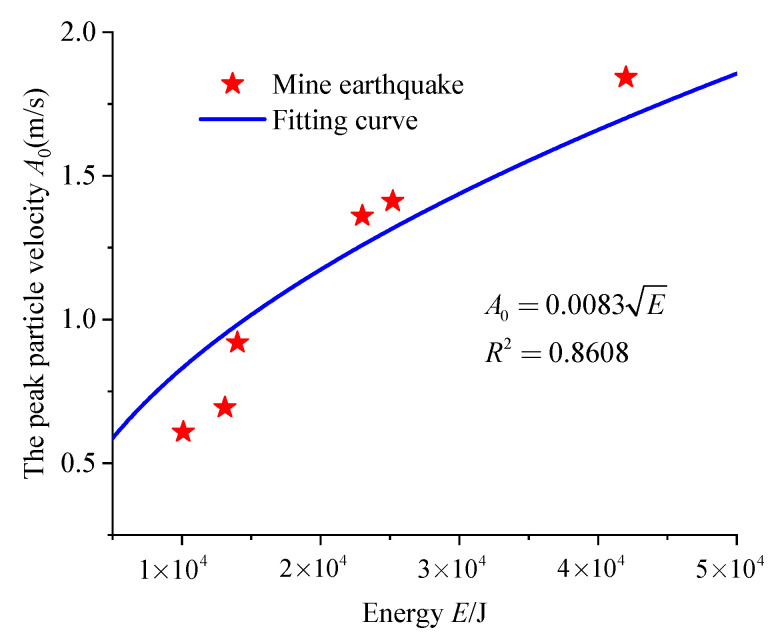
Mine earthquake amplitude and energy relation fitting.

After data processing and analysis, the PPV attenuation model and model parameters for each individual mine earthquake listed in Table 2 were obtained based on the seismic wave propagation law. According to the attenuation model, the attenuation curve, as shown in Figure 8, is drawn. It can be seen in Figure 8 that the models satisfactorily carried out the fitting of the amplitude values recorded by various sensors. Additionally, the correlation coefficients are all greater than 0.8, indicating that the fitting effect is good. Figure 9 shows the fitting relationship between peak particle velocity and energy of the mine earthquake at the source.

Based on the analysis above, the fitting coefficient *C* between the source energy and amplitude was identified as 0.0083, and the attenuation coefficient *α*_0_ was 0.0020. Thus, the roadway disaster-causing model for the 37221-1 upper roadway of the Dongxia Coal Mine was established as follows:

(10) $$E=\frac{A_{P}{}^{2}}{6.89\times 10^{-5}}r^{2}e^{0.0040r}$$

### 3.3. Critical PPV Determined by Numerical Simulation

#### 3.3.1. Model Establishment

**Figure 10:**
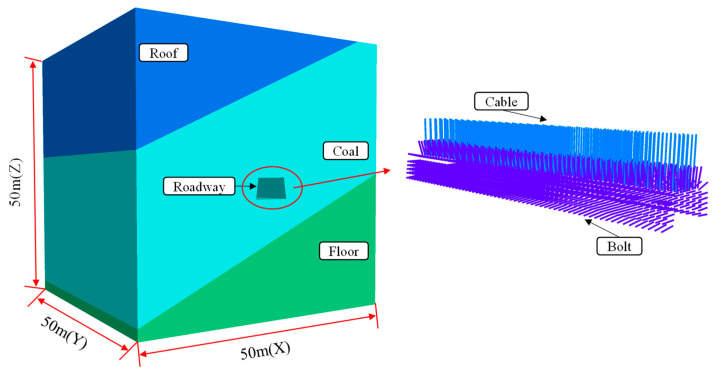
Numerical model.

In the numerical analysis herein, FLAC^3D^ software was adopted to establish the simplified three-dimensional numerical model designed as 50 m × 50 m × 50 m (length × width × height), as shown in Figure 10, based on the geological environment, roadway deployment, and support conditions around the Dongxia Coal Mine 37221-1 working face. Besides, the dip angle of the coal seam is 25°, and the cross section of the roadway is a trapezoid that is 5400 mm at the top, 6400 mm at the bottom, and 3700 mm high. Thanks to the burial depth and significant tectonic geological environment where the roadway is located, the stress applied to the top and surroundings of the model were 12.50 MPa and 18.75 MPa, respectively. At the same time, the structural unit ‘Cable’ was adopted to simulate bolts and cables, and integrated into the model as well. Table 3 shows the physical and strength parameters of every structural unit.

To ensure the accuracy of experimental results and rock mechanics’ parameters during the numerical simulation, the rock mechanics’ parameters involved in all the numerical simulation and theoretical calculation and analysis in this paper were obtained through the estimation on intact rocks based on the generalized Hoek–Brown failure criterion [52]. The physical and mechanical parameters of the coal seam are shown in Table 4.

In addition, for deep-buried roadway, relevant experimental and numerical simulation studies show that the existence of ground stress will affect the propagation velocity of vibration waves in the rock mass [53,54]. Therefore, the initial ground stress must be balanced before dynamic load is applied.

#### 3.3.2. Influence of Dynamic Load on Deformation of Rocks Surrounding Roadway

**Figure 11:**
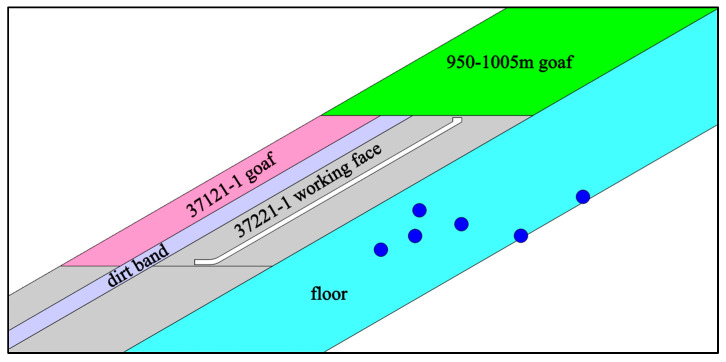
Projection diagram of mine earthquake cross section.

After the high energy mine earthquakes were located, it was found that they mostly gathered 50 m below the roadway of the 37221-1 working face, as shown in Figure 11. Therefore, dynamic load was designed to be 50 m below the roadway, i.e., 30 m below the model. Meanwhile, dynamic load was applied by time-step based on the distance from the bottom grid of the model to the source and presumed to propagate at 4100 m/s outside the model (equal to the propagation velocity of P waves used for location). Based on the simulation and characterization methods of roadway failure adopted in this paper, the failure deformation of various parts of the roadway are shown in Table 5.

In engineering practice, the deformation of coal and rock mass is related to its constitution. When dynamic load propagates to coal and rock mass via rock mass, stress effect will be reduced, but the deformation of coal and rock mass and the peak particle velocity of dynamic load will both be increased. In the meantime, the free face formed after the excavation of the roadway reflects part of the propagated dynamic load, generating the phenomenon of wave superposition, and increases the peak particle velocity near the roadway to a level even higher than the velocity of the applied dynamic load. In the numerical simulation herein, six groups of PPV simulation and test plans were set up, with PPVs as the simulation variables, and similar results as described above were obtained by monitoring the peak particle velocity on the roadway surface. The test plans and monitoring results are shown in Table 6.

**Figure 12:**
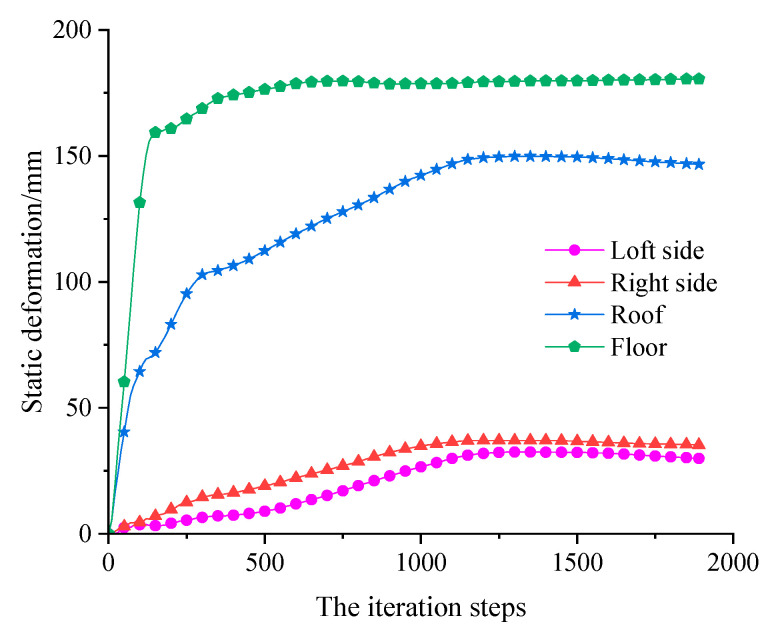
Deformation of roadway under static load.

**Figure 13:**
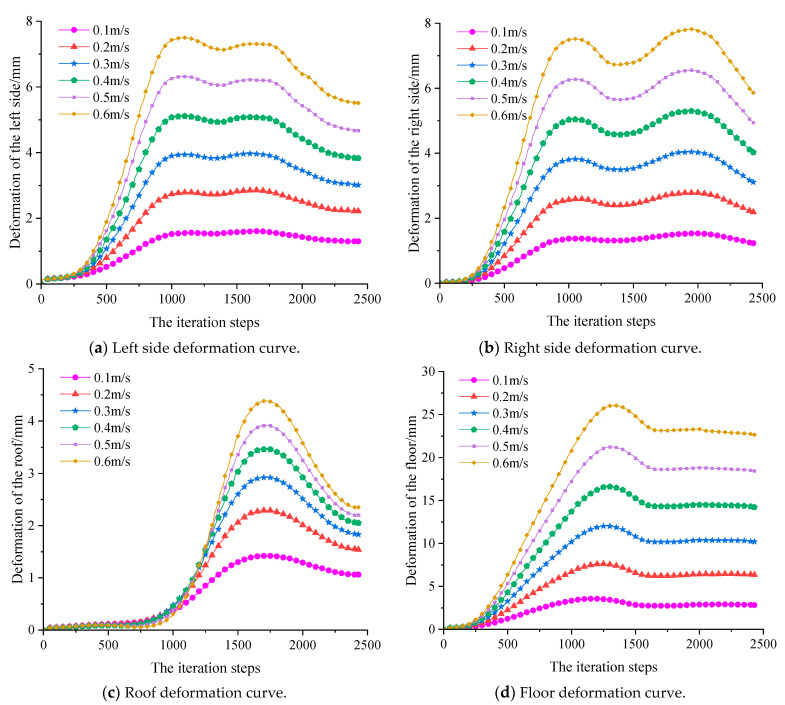
Roadway deformation curve with respect to different PPVs.

**Figure 14:**
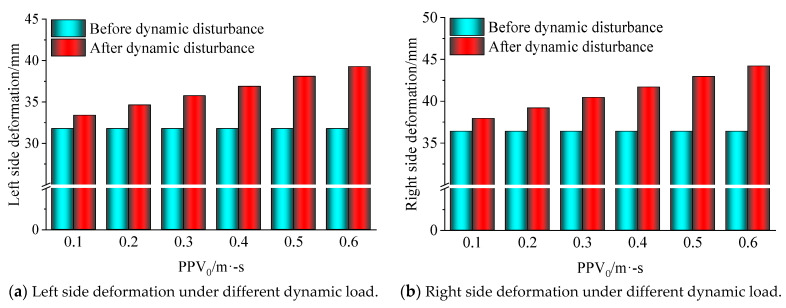

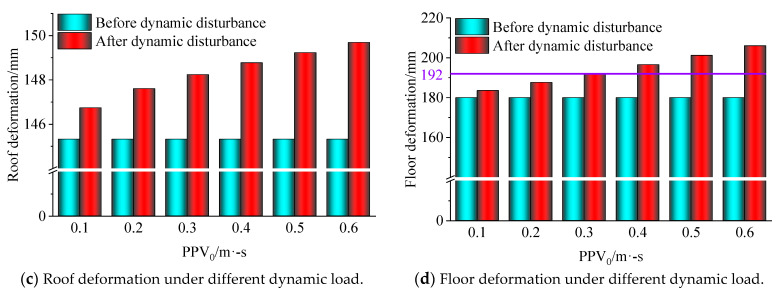
Roadway deformation with respect to different PPVs.

Meanwhile, displacement monitoring points are also arranged around the roadway to monitor the deformation of the roadway surface. Among them, the deformation of roadway surface under static load is shown in Figure 12, and the deformation curve of roadway surface after dynamic load with different amplitudes is shown in Figure 13. After superimposing the maximum displacement around the roadway under static and dynamic loading, the deformation of the roadway under different PPVs as shown in Figure 14 can be obtained.

According to Figure 14a,b, the deformation of the both sides of the roadway was relatively low under static load, but increased after they were disturbed by dynamic load. When the dynamic load reached 0.6 m/s, the deformation of the both sides went up to 40 mm and 45 mm, respectively, which were still far from enough to cause roadway failure.

Figure 14c,d show the deformation of the roof and floor of the roadway under static load and dynamic load. It can be seen in the figure that the floor deformation under static load is higher than the roof deformation mainly because the bolts and cables installed in the roof provided effective support and the horizontal stress caused larger deformation to the floor. Besides, the deformation of the roof was increased less upon dynamic load disturbance, and only increased by 7.8 mm even if the applied dynamic load reached 0.6 m/s; still far from enough to cause roof failure. On the contrary, the deformation of the floor was more increased upon dynamic load disturbance, and reached 192 mm under the combined static and dynamic load when the applied dynamic load was 0.3 m/s. At that moment, the PPV on the roadway surface was 0.389 m/s, which can be deemed as the failure of the roadway floor.

Based on the simulation results and analysis above, it can be concluded that the critical resistible PPV *A_P_* for the floor of the 37221-1 working face upper roadway of the Dongxia Coal Mine is about 0.4 m/s, i.e., the roadway disaster-causing model of the Dongxia Coal Mine can be expressed as:

(11) $$E=2.32\times 10^{3}r^{2}e^{0.0040r}$$

### 3.4. Anti-Burst Performance Evaluation and Model Verification

**Figure 15:**
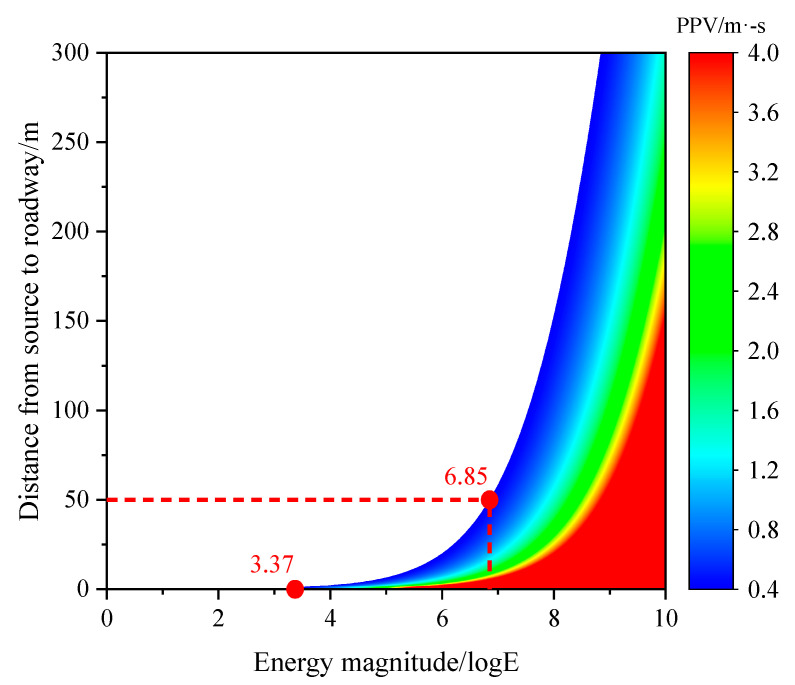
PPV contour cloud diagram of roadway disaster-causing model.

The contour cloud diagram of PPV distribution shown in Figure 15 was drawn using the roadway disaster-causing model expressed in Equation (11). According to the diagram, PPV does not exceed 0.4 m/s, the critical burst velocity of the roadway identified via simulation, when the magnitude of the mine earthquake energy is lower than 3.37, and thus the existing roadway support system of the Dongxia Coal Mine 37221-1 working face upper roadway is able to resist mine earthquakes with energy lower than 10^3.37^ J, i.e., 2.33 × 10^3^ J, however far the source is from the roadway. The volume of surrounding rocks required for energy accumulation becomes obviously larger with the increase in mine earthquake energy. Normally, the fracture radius of the mine earthquake with high energy is over 50 m. Additionally, the effect of destress measures are found in the strata close to the coal seam. Therefore, the maximum resistible mine earthquake energy is 10^6.85^ J, i.e., 7.09 × 10^6^ J, when the roadway is 50 m from the source, with both the energy accumulation volume of surrounding rocks and the fracture range of the source considered.

To verify the effectiveness of the proposed method, model verification was conducted using two cases of severe rockburst occurring during the stoping of the 37221-1 working face. The source parameters solved by the SOS micro-seismic monitoring system and the critical burst energy, *E*′, obtained via the disaster-causing model are shown in Table 7. It can be seen that the actual burst energies, *E*, of the two rockburst cases are both close to the burst-induced critical energy, *E*′, indicating that the disaster-causing model established in this paper features good applicability and can accurately evaluate the anti-burst performance of a roadway support system.

## 4. Discussions

With the coal resource excavation gradually going deeper, the excavation environment on the working face has become more complex and the risk of rockburst has therefore increased. This is the reason why higher requirements are required for the performance of roadway support and a method is needed for scientific evaluation of the anti-burst performance of roadway support systems. The evaluation method of the roadway support system performance proposed in this paper overcame the problem that traditional verification methods are inapplicable for dynamic load and provided a new idea for the research into roadway support. Meanwhile, this paper has some issues that need further discussing and researching.

The selection of mine earthquakes is one of the key factors that affect the establishment of mine earthquake attenuation models. The mine earthquakes used to establish the models in this paper were near the roadway and featured high energy. Most of the mine earthquake events during excavation feature low energy, but mine earthquake with low energy are also significant for roadway rockburst as long as their number is sufficient. It is worthy of deep consideration how to reasonably identify the number of mine earthquake events to be used for the establishment of mine earthquake attenuation models and whether the number of selected mine earthquakes need to correspond to a certain range of energy. At the same time, due to the difference of constitution between coal and rock, the seismic wave signals received by the sensors installed in a coal seam are different from those in a stratum. Therefore, how to establish specific coal-seam–stratum signal conversion relationship based on the stratum characteristics of a coal mine is of importance to the establishment of more improved attenuation models. Besides, since the coal mines are currently using small-range sensors and the received signals often exceed the range of the sensors when they are installed close to the source or in the coal seam, it is significant to consider how to identify reasonable PPV using algorithms and fitting that are more scientific. Finally, specific disaster-causing models shall be established for coal mines and roadways of different types, and they shall be sufficiently verified regarding their effectiveness based on multiple historic cases of rockburst and continuously improved with respect to their applicability.

## 5. Conclusions

(1) Based on the propagation law of peak particle velocity, this paper selected the mine earthquakes near the 37221-1 upper roadway of the Dongxia Coal Mine, studied the attenuation characteristics of the seismic waves, and established the roadway disaster-causing model.

(2) According to the simulation results, the tectonic stress in the Dongxia Coal Mine and the support effect of the roadway roof and sides jointly caused the deformation of the roadway floor to be most significant under the combination of dynamic and static loads. When the PPV near the roadway reached 0.4 m/s or so, failure first occurred in the roadway floor.

(3) The PPV contour cloud diagram was drawn based on the established roadway disaster-causing model, and could be used to obtain the burst-induced critical energy of the mine earthquakes at locations with different distances to the 37221-1 upper roadway. The effectiveness and applicability of the disaster-causing models were verified using two cases of severe rockburst that occurred during the 37221-1 face retreat.

## Figures and Tables

**Table 1:** Classification and comparison of squeezing deformation behaviors [48,49,50,51].

Classification No.	Aydan		Singh		Hoek	
	Deformation Degree	Relative Displacement%	Deformation Degree	Relative Displacement%	Deformation Degree	Relative Displacement%
1	No squeezing deformation	≤1.0	—	—	Stable	≤1.0
2	Slight squeezing deformation	1.0–2.0	Slight squeezing deformation	1.0–3.0	Slight squeezing deformation	1.0–2.5
3	Medium squeezing deformation	2.0–3.0	Medium squeezing deformation	3.0–5.0	Medium squeezing deformation	2.5–5.0
4	Severe squeezing deformation	3.0–5.0	Severe squeezing deformation	>5.0	Severe squeezing deformation	5.0–10.0
5	Extremely severe squeezing deformation	>5.0	—	—	Extremely severe squeezing deformation	>10.0

**Table 2:** Mine earthquake attenuation models.

Number	Mine Earthquake Attenuation Models	The PPV at the Source *A*_0_ (m/s)	Energy *E*(J)	Attenuation Coefficient *α*
1	$A=0.6086\frac{1}{r}e^{-0.0016r}$	0.6086	1.01 × 10^4^	0.0016
2	$A=0.6939\frac{1}{r}e^{-0.0009r}$	0.6939	1.31 × 10^4^	0.0009
3	$A=1.4117\frac{1}{r}e^{-0.0048r}$	1.4117	2.52 × 10^4^	0.0048
4	$A=2.1077\frac{1}{r}e^{-0.0019r}$	2.1077	4.20 × 10^4^	0.0016
5	$A=1.3603\frac{1}{r}e^{-0.0019r}$	1.3603	2.30 × 10^4^	0.0019
6	$A=0.9199\frac{1}{r}e^{-0.0010r}$	0.9199	1.40 × 10^4^	0.0010

**Table 3:** Support structure parameters.

Type	Bolt Length/mm	Grouting Length/mm	Diameter/mm	Tensile Strength/kN	Pretightening Force/kN	Row-to-Row Space/mm
Bolt	2600	1200	22	190	150	700 × 800
Cable	6300	1900	22	710	280	1000 × 800

**Table 4:** Mechanical parameters of coal seam and rock layers.

Lithology	Bulk Modulus /GPa	Shear Modulus /GPa	Cohesion /MPa	Internal Friction Angle (°)	Tensile Strength/MPa	Density /kg·m^−3^
Roof	3.67	2.20	6.24	35	0.47	2400
Coal seam	0.96	0.33	1.20	28	0.14	1400
Floor	2.50	1.15	4.00	33	0.21	2200

**Table 5:** Deformation of roadway failure.

Location	Roadway Size/mm	Relative Failure Deformation	Failure Deformation/mm
Roof	5400	3%	162
Floor	6400	3%	192
Both sides	3734	3%	112

**Table 6:** Model design with different PPVs.

Model	PPV_0_/(m·s^−1^)	PPV_1_/(m·s^−1^)	PPV_2_/(m·s^−1^)	PPV_3_/(m·s^−1^)	PPV_4_/(m·s^−1^)
1	0.10	0.066	0.067	0.051	0.130
2	0.20	0.128	0.133	0.093	0.259
3	0.30	0.181	0.195	0.126	0.389
4	0.40	0.229	0.248	0.156	0.520
5	0.50	0.278	0.297	0.183	0.650
6	0.60	0.327	0.352	0.210	0.779

PPV_0_: PPV of applied dynamic load; PPV_1_: PPV of applied dynamic load propagated to the left side of roadway; PPV_2_: PPV of applied dynamic load propagated to the right side of roadway; PPV_3_: PPV of applied dynamic load propagated to the roof of roadway; PPV_4_: PPV of applied dynamic load propagated to the floor of roadway.

**Table 7:** Mine earthquake source parameters and burst-inducing critical energy.

Mine Earthquake	Distance from Sourceto Roadway/m	Calculated Energy *E*/J	Burst-Inducing Critical Energy *E*′/J
1	17.6	7.05 × 10^5^	7.72 × 10^5^
2	14.4	5.71 × 10^5^	5.10 × 10^5^

## Data Availability

The seismic data used in this work are from Dongxia Coal Mine, and they are confidential.
